# Integrated Genomic and Transcriptomic Analysis Reveals a Transcription Factor Gene Set Facilitating Gonadal Differentiation in the Pacific Oyster *Crassostrea gigas*

**DOI:** 10.3390/genes16050513

**Published:** 2025-04-28

**Authors:** Yunwang Shen, Ziyi Wang, Yanglei Jia, Xiao Liu

**Affiliations:** 1 National Engineering Research Center for Marine Aquaculture, Zhejiang Ocean University, Zhoushan 316022, China; shenyw@zjou.edu.cn (Y.S.); 13958691475@163.com (Z.W.); 2 Zhoushan Fishery Breeding and Hatching Innovation Center, Zhejiang Ocean University, Zhoushan 316022, China

**Keywords:** Pacific oyster, *Crassostrea gigas*, transcription factor, sex determination, gonadal differentiation

## Abstract

Background/Objectives: The Pacific oyster *Crassostrea gigas* has emerged as a promising model system for sex determination studies due to its complex reproduction strategy and sex reversal. Transcription factors (TFs) play crucial roles in sex determination and gonadal differentiation. Despite previous research revealing functions of several conserved sex-determining pathway genes, such as *Dmrt1*, *Foxl2*, and *SoxH*, little is known about the other essential TF regulators driving *C. gigas* gonadal differentiation and development. Methods: In this study, a systematic identification of TFs revealed 1167 TF genes in the *C. gigas* genome. Comparative transcriptome analysis of *C. gigas* female and male gonads demonstrated 123 differentially expressed TF genes. Results: The majority of these sex-related TF genes were up-regulated in female or male gonads from the inactive stage to the mature stage. Moreover, this TF gene set was deeply conserved and showed similar regulation in the Kumamoto oyster *Crassostrea sikamea* gonads, suggesting their important regulatory roles in gonadal differentiation and development in Crassostrea oysters. Furthermore, two BTB TF gene clusters were identified in the *C. gigas* genome, both of which were specifically expressed in the male gonad. Gene numbers of each BTB gene cluster showed significant variations among six Crassostrea species. Conclusions: To the best of our knowledge, this study provides the first report of the whole TF family in *C. gigas*. The sex-related TF gene set will be a valuable resource for further research aimed at uncovering TF gene regulatory networks in oyster sex determination and gonadal differentiation.

## 1. Introduction

The Pacific oyster *C. gigas*, also referred to as *Magallana gigas*, has been one of the most popular aquaculture species for food source around the world [1]. In recent years, *C. gigas* has emerged as a good model organism for sex determination studies due to its complex reproduction strategies. It is generally believed that Pacific oysters are protandric hermaphrodites in which a majority of one-year-old oysters would develop into males but change to females in later years [2]. Moreover, their sex ratios are also influenced by environmental factors, such as temperature, nutrient supply, and environmental pH [3,4,5]. Generally, high temperature, sufficient food resources, and low pH could induce more females in Pacific oysters. Therefore, sex determination of *C. gigas* is controlled by both genetic and environmental factors, in which their interaction mechanisms remain unknown [6]. The rhythmical and occasional sex reversal phenomena in *C. gigas* populations make their reproduction strategy and sexual differentiation much more complicated [7,8]. From the perspective of gametogenesis, female and male individuals could be distinguished by the process of oogenesis and spermatogenesis, respectively. Both female and male gonads follow an annual reproductive cycle of five developmental stages: inactive, formative, proliferative, mature, and proligerous [9]. Numerous studies have been focused on the *C. gigas* sex-determination mechanism from the genomic, transcriptomic, proteomic, and epigenomic levels [8,10,11]. Despite several sex-related genes having been characterized, the complex sexual system of *C. gigas* remains elusive.

Transcription factors (TFs) play pivotal roles in regulating chromatin state and gene transcription through binding specific DNA sequences [12]. TFs are involved in almost all kinds of biological processes, such as cell differentiation, tissue development, and immune responses [12,13,14]. The past two decades have witnessed the outbreak of TF investigations in bivalves, especially in the Pacific oyster. Doublesex and mab-3-related transcription factor 1 (*Dmrt1*) and forkhead box L2 (*Foxl2*), the conserved sex-determining genes, are crucial TF regulators in *C. gigas* gonadal differentiation and maintenance [6,15,16]. Relative expression levels of *Dmrt1* and *Foxl2* in *C. gigas* gonads are able to determine the directions of gonadal differentiation (female or male), suggesting an antagonistic regulatory effect of the two TFs [16]. In addition, an increasing number of studies have revealed the important roles of the Sox gene family in *C. gigas* sex-determining pathways [17,18,19,20,21]. *Sox-like* and *SoxH* are predominantly expressed in mature male gonads [19,20], while *SoxB1* is specifically expressed in mature female gonads [20]. *Sohlh1* and *Sohlh2*, two members of the bHLH family, are inversely expressed in *C. gigas* female and male gonads [22]. However, these TFs are usually predicted based on similarities with their well-studied homologues in mammalian and invertebrate model organisms. Our understanding of the TF gene regulation networks in oyster gonads is still insufficient.

To date, it remains to be resolved how many TFs are involved in *C. gigas* gonadal differentiation and development. To this end, in the current research, 1167 TF genes were systematically identified in the *C. gigas* genome. Furthermore, comparative transcriptome analysis of *C. gigas* female and male gonads revealed a gene set of 123 TFs that were related to gonadal differentiation and development. This TF gene set contained well-known conserved sex-determining pathway genes, including *Dmrt1*, *Foxl2*, and Sox genes. Most of these TFs showed similar regulation in the Kumamoto oyster *C. sikamea* gonads, indicating their conserved regulatory roles in the Crassostrea species. In addition, two BTB gene clusters were identified in the genome that were predominantly expressed in male gonads. Both of the gene clusters showed significant gene number variations among six Crassostrea species (*C. gigas*, *C. angulata*, *C. ariakensis*, *C. hongkongensis*, *C. nippona*, and *C. sikamea*). Importantly, the functions of the most sex-related TF genes remain to be elucidated. Taken together, this work provides the first report of genome-wide identification of the whole TF family in *C. gigas*. The results will facilitate our understanding of TF gene regulatory networks in Crassostrea gonadal differentiation and development. The sex-related gene set of 123 TFs will provide a valuable resource for future studies aimed at uncovering TF-mediated regulation of sex determination and sex reversal.

## 2. Materials and Methods

### 2.1. Identification of Transcription Factors Based on Pacific Oyster Genome

The Pacific oyster *C. gigas* genome (accession number: GCF_902806645.1) and annotation files were downloaded from NCBI. This *C. gigas* chromosome-level assembly contains 10 pseudo-chromosomes and 226 unplaced scaffolds [1]. A total of 63,341 protein sequences are annotated in the genome, corresponding to 31,371 genes (Supplementary Table S1). Transcription factors (TFs) were identified using the ‘Predict TF’ module of AnimalTFDB v4.0 (https://guolab.wchscu.cn/AnimalTFDB4/#/TF_Predict, accessed on 10 March 2025). Then, *C. gigas* TFs were classified into 73 families based on their DNA-binding domain (DBD) types. The genome GFF file was used to analyze TF chromosomal localization. TF alternative splicing events were identified by Astalavista (v4.0), including exon skipping, alternative 5′ splicing site, alternative 3′ splicing site, intron retention, and mutually exclusive exons. Sashimi plots of TF alternative splicing sites were visualized in Integrative Genomics Viewer (IGV) software (v2.8.0) based on *C. gigas* gonadal transcriptomes that will be described in detail below.

### 2.2. Expression Pattern Analysis of Pacific Oyster Transcription Factors in Adult Tissues

The Pacific oyster transcriptomes of nine adult tissues were downloaded from the NCBI Sequence Read Archive (SRA) database, including adductor muscle, digestive gland, male gonad, hemolymph, female gonad, gill, labial palp, mantle outer region, and mantle inner region. Their accession numbers are listed in Supplementary Table S2. Reads with poly-N and low-quality reads were removed from raw data using Trim Galore (v0.6.7). Clean data were aligned to the *C. gigas* genome (GCF_902806645.1) using HISAT2 (v2.2.1) and were converted into BAM files by SAMtools (v11.6). Fragments per kilobase of transcript per million fragments mapped (FPKM) values were generated by StringTie (v2.2.1) to quantify gene expression levels. In this study, genes with FPKM ≥ 1 were considered to be expressed in the tissue.

### 2.3. Comparative Transcriptome Analysis of Pacific Oyster Female and Male Gonads

Two transcriptome datasets of *C. gigas* female and male gonads were downloaded from the NCBI SRA database. These data are from two studies investigating the *C. gigas* sex-determining mechanism [10,23]. In this study, the two datasets were referred to as the “F2n_vs_M2n” group and the “s3F_vs_s3M” group, respectively. Both groups compare transcriptional differences between female and male gonads in the mature stage. Transcriptome data of *C. gigas* gonads in the inactive stage (termed as s0) were also analyzed to investigate dynamic gene expression during gonadal maturation in *C. gigas* (Supplementary Table S2). Raw data were processed, and gene expression levels were investigated as described in Section 2.2. For differentially expressed gene (DEG) analysis between female and male gonads, gene counts were generated using FeatureCounts (v2.0.1) based on BAM files. DEGs were identified by use of the R package DESeq2 (v1.46.0) with a false discovery rate (FDR) < 0.01 and fold change ≥ 2 or ≤0.5. DEGs with average FPKM values < 1 in both sexes were removed. The shared up- and down-regulated DEGs in the two datasets were used for KEGG enrichment analyses.

### 2.4. De Novo Transcriptome Analysis of Kumamoto Oyster Gonads

Kumamoto oyster (*Crassostrea sikamea*) is a common oyster species along the intertidal zone of Zhejiang Province [24]. Wild *C. sikamea* individuals were collected in Shengsi, Zhoushan. Female and male gonads in the mature stage were distinguished and sampled for transcriptome sequencing. Four biological replicates were prepared for each sex (Supplementary Table S2). Eight RNA-Seq libraries were constructed in a routine procedure [25] and were sequenced on an Illumina Novaseq 6000 platform with 150 bp paired-end (PE) reads model. After removing reads with poly-N and low-quality reads, the de novo transcriptome was assembled by Trinity (v2.13.2). FPKM values of unigenes were generated using eXpress (v1.5.1). Bowtie2 (v2.3.3.1) was used to obtain the number of reads aligned to unigenes in each sample. Then gene counts were used for DEG identification by DESeq2 with a false discovery rate (FDR) < 0.05 and fold change ≥ 2 or ≤0.5. To further compare the TF gene regulation between *C. gigas* and *C. sikamea*, diamond (v2.0.12) blastp was used to find homologues in the *C. sikamea* transcripts using *C. gigas* TF sequences as the query.

### 2.5. Reverse Transcription PCR (RT-PCR) and Quantitative Real-Time PCR (qRT-PCR) Analyses

Total RNA was extracted from *C. sikamea* female and male gonads using Trizol (Sangon Biotech, Shanghai, China) following the manufacturer’s instructions. The quantities and qualities of RNA samples were examined using NanoDrop 2000 (ThermoFisher Scientific, Waltham, USA). About 1 μg of RNA from each sample was converted into cDNA using the Evo M-MLV Plus 1st Strand cDNA Synthesis Kit (Accurate Biotechnology, Changsha, China) in a 20 μL volume. The cDNA samples were diluted into two-fold volumes. Then, 0.5 μL diluted cDNA was used as a template for RT-PCR analysis. The qRT-PCR experiments were conducted in three biological replicates using the same cDNA samples. Then cycle threshold (CT) values were used to calculate fold change according to the 2^−ΔΔCT^ method [26]. For both RT-PCR and qRT-PCR analyses, the elongation factor 1-α (EF1α) was used as the reference gene. Primer sequences and the corresponding *C. gigas* homologues used for primer design are listed in Table 1.

### 2.6. BTB Gene Duplication Analyses in Crassostrea Species

Nine genome assemblies of five Crassostrea species were downloaded from NCBI, including *C. gigas*, *C. angulata*, *C. ariakensis*, *C. hongkongensis*, and *C. nippona*. Homologues of the two BTB gene clusters (BTBGCs) were screened in these genomes by blastn (v2.13.0). Considering partial gene fragment loss during gene duplication, the largest exon (exon 2 for BTBGC1 and exon 3 for BTBGC2) was used as the query to find identical or similar DNA sequences in the genome. BTB gene duplication was also evaluated in multiple *C. sikamea* individuals by PCR. Total genomic DNA was extracted from *C. sikamea* adductor muscle samples using the SteadyPure Universal Genomic DNA Extraction Kit (Accurate Biotechnology, Changsha, China) according to the manufacturer’s instructions. Eight *C. sikamea* individuals were randomly selected for PCR analysis. Two pairs of primers were designed to amplify genomic fragments spanning two exons of BTBGC1 (LOC117688191 as a representative) and BTBGC2 (LOC105330609 as a representative). *C. sikamea* gonadal cDNA samples were also used as templates to investigate the expression of the two BTBGCs, as described in Section 2.5. Primer sequences of BTBGC1 and BTBGC2 are listed in Table 1.

## 3. Results

### 3.1. Overview of Transcription Factors in the Pacific Oyster Genome

**Figure 1:**
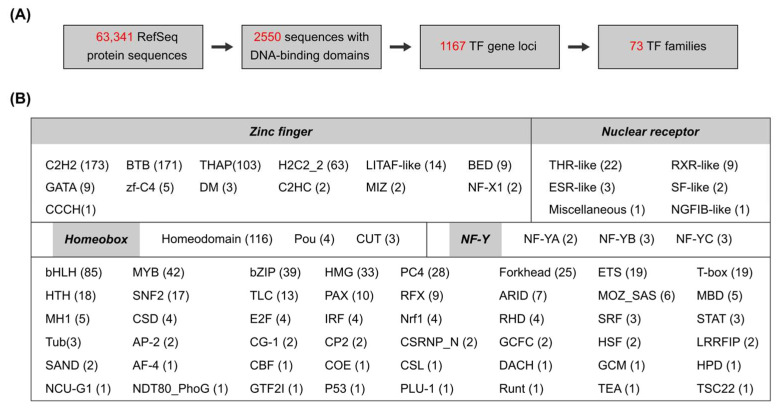
Genome-wide identification of Pacific oyster transcription factors. (**A**) Schematic diagram of TF identification based on *C. gigas* genome and annotations. (**B**) Summary of TF families and their gene numbers in *C. gigas*.

The reference genome of the Pacific oyster *C. gigas* (NCBI accession number GCF_902806645.1) was used to systematically identify transcription factor (TF) genes. The genome contains 63,341 annotated proteins in 31,371 gene loci (Supplementary Table S1). By use of the AnimalTFDB v4.0 database [13], 2550 sequences were predicted to contain at least one DNA-binding domain (DBD), corresponding to 1167 genes (Figure 1A). These 1167 genes were further classified into 73 TF families based on their DBD types (Supplementary Table S3). TFs with more than one type of DBD were classified into one certain family according to the rules described on the AnimalTFDB website (https://guolab.wchscu.cn/AnimalTFDB4_Document/AboutTFfamily/, accessed on 10 March 2025).

Notably, the zinc finger and homeobox families constituted the majority of *C. gigas* TFs, accounting for 47.56% and 10.54% of total TF genes, respectively (Figure 1B). About 91.89% of zinc finger TFs were classified into four families: C2H2 (173), BTB (171), THAP (103), and H2C2_2 (63). In addition, 38 nuclear receptor TFs were predicted in the *C. gigas* genome, in which thyroid hormone receptor-like (THR-like) was the most abundant family. *C. gigas* was also enriched in basic helix–loop–helix (bHLH), MYB, basic leucine zipper (bZIP), high mobility group (HMG), PC4, and Forkhead TF families, all of them containing more than 20 genes.

**Figure 2:**
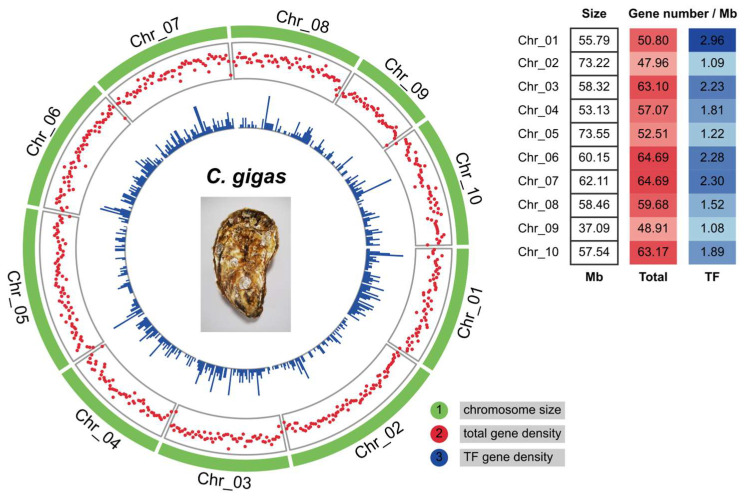
Distribution of transcription factor genes on ten chromosomes in *C. gigas*. Circos plots showed chromosome size, total gene, and TF gene density from outer to inner circles. The sliding window size was set as 1 Mb. The right table shows the average gene number per Mb sequence on ten *C. gigas* chromosomes.

Furthermore, the chromosomal localization of *C. gigas* TFs was investigated. Circos plots showed that *C. gigas* TFs were unevenly distributed on ten chromosomes (Figure 2). Chr_02 (NC_047560.1) and Chr_09 (NC_047567.1) showed the scarcest TF gene densities, with on average only one TF gene per million base pair (Mb) sequence. On the contrary, Chr_01, Chr_06, and Chr_07 were enriched in TF genes. Chr_02 is the second-largest chromosome in *C. gigas*. However, it contained fewer genes from the point of gene number (about 47.96 total genes and 1.09 TF genes) per Mb sequence. Gene densities of four major TF families (gene number > 100) were further analyzed within each chromosome, including C2H2, BTB, THAP, and homeobox (homeodomain, CUT, and POU). It seemed that a part of the TF genes in the four families was distributed in gene clusters on certain chromosomes (Supplementary Figure S1A). Chr_02 contained 17 C2H2, 16 BTB, and 21 THAP genes, while no homeobox gene was found in this chromosome. Chr_09 is the smallest chromosome in *C. gigas* (Figure 2) and contains a small number of TF genes (Supplementary Figure S1A). In addition, multiple BTB gene clusters were observed in Chr_02, Chr_07, and Chr_08 (Supplementary Figure S1B). For example, nine BTB genes (from LOC105338015 to LOC117688191) were located within the 0.5 Mb sequence on Chr_02.

### 3.2. Alternative Splicing of Pacific Oyster Transcription Factors

**Figure 3:**
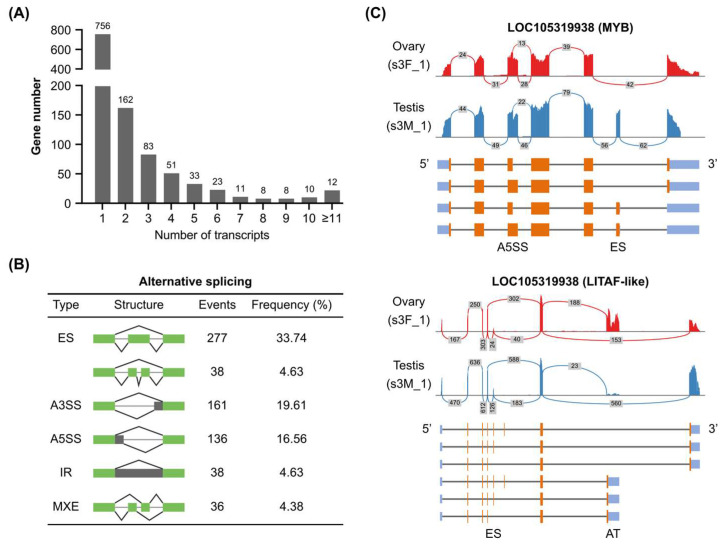
Alternative splicing profiles of Pacific oyster transcription factors. (**A**) Summary of transcript numbers of *C. gigas* TF genes. (**B**) Alternative splicing types in *C. gigas* TF genes. (**C**) Sashimi plots of two TF genes with multiple alternative splicing sites. Numbers in the grey rectangles indicated RNA-seq reads aligned to the junction spanning the exons. ES, exon skipping; A3SS, alternative 3′ splicing site; A5SS, alternative 5′ splicing site; IR, intron retention; MXE, mutually exclusive exons; AT, alternative terminator.

Alternative splicing, the process of generating multiple splice isoforms from a single gene, contributes significantly to the complexity of eukaryotic transcriptomes [27]. LOC105345687 (Homeodomain) was annotated with 50 transcripts in the *C. gigas* genome (Supplementary Table S3). Based on the genome annotation, about 34.66% (401 out of 1157) of *C. gigas* TF genes contained alternative splicing (Figure 3A) with an average of 4.47 isoforms per gene. It was noticed that some TF families preferred to produce alternative spliced transcripts, such as GATA, THR-like, HMG, and ETS (Supplementary Figure S2A). Alternative splicing contains five different types, including ES (exon skipping), A3SS (alternative 3′ splicing site), A5SS (alternative 5′ splicing site), IR (intron retention), and MXE (mutually exclusive exons). A total of 821 alternative splicing events were found in *C. gigas* TF genes, in which exon skipping was the most abundant type (Figure 3B). In addition, *C. gigas* TF alternative splicing showed tissue-specific or cell-type-specific expression patterns. For example, the ES event of LOC105335652 (GATA) was found in both female and male gonads (Supplementary Figure S2B). However, the ES event of LOC105319938 (MYB) was specifically found in the female gonad (Figure 3C). Some TF genes contained more than one type of alternative splicing. LOC105319938 (LITAF-like) possessed both A5SS at the third exon and ES at the sixth exon, making it four potential isoforms (Figure 3C). Besides the five main alternative splicing types, alternative promoters and alternative terminators were also found in *C. gigas* TF genes. LOC105319938 and LOC105325112 (HMG) could produce isoforms with different C-terminal residues (Figure 3C and Supplementary Figure S2B). Importantly, a C-terminal isoform of LOC105325112 was specifically expressed in the male gonad (Supplementary Figure S2B). Taken together, alternative splicing and tissue-specific expression contributed to the complex transcripts of *C. gigas* TFs.

### 3.3. Expression Patterns of Transcription Factors in Pacific Oyster Adult Tissues

**Figure 4:**
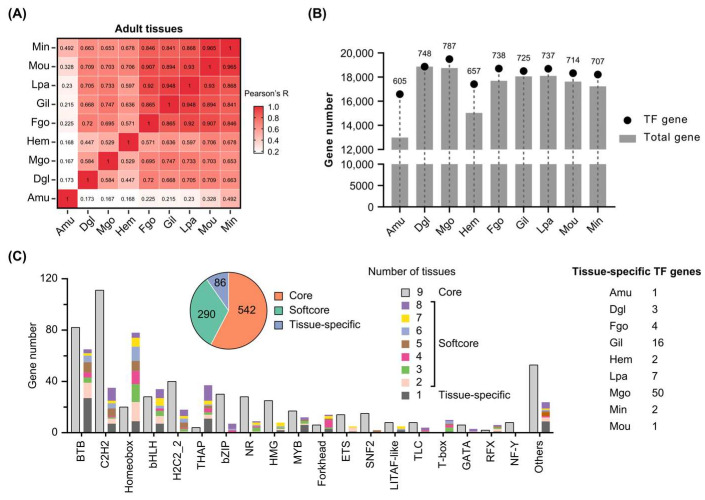
Expression patterns of Pacific oyster transcription factors in adult tissues. (**A**) Pearson correlation coefficient of nine adult tissues based on total gene expression levels. (**B**) Number of total genes and TF genes expressed in nine tissues. (**C**) Diverse expression patterns of TF families in adult tissues. *C. gigas* TFs were classified into three groups: core, softcore, and tissue-specific. Amu: adductor muscle, Dgl: digestive gland, Mgo: male gonad, Hem: hemolymph, Fgo: female gonad, Gil: gill, Lpa: labial palp, Mou: mantle outer region, Min: mantle inner region.

Next, expression levels of *C. gigas* TFs were examined in adult tissues based on transcriptomes, including the adductor muscle (Amu), digestive gland (Dgl), male gonad (Mgo), hemolymph (Hem), female gonad (Fgo), gill (Gil), labial palp (Lpa), mantle outer region (Mou), and mantle inner region (Min). Pearson correlation coefficient based on total gene expression levels revealed that the adductor muscle was significantly different from the other tissues, sharing 16.8%~49.2% similarities (Figure 4A). Female and male gonads shared 69.5% similarity. Based on FPKM value ≥ 1, the adductor muscle expressed much fewer total and TF genes than the other tissues, while the male gonad expressed the largest number of TF genes (Figure 4B).

A total of 918 TF genes were expressed in nine adult tissues, accounting for 78.66% of *C. gigas* TFs (Supplementary Table S4). These TFs were divided into three categories: core, softcore, and tissue specific. Core TF genes were ubiquitously expressed in the nine tissues. Softcore TF genes were expressed in at least two but not all tissues. Tissue-specific TF genes were expressed in only one type of the nine tissues. Finally, 542 core, 290 softcore, and 86 tissue-specific TFs were identified in *C. gigas* (Figure 4C). Male gonads expressed the most tissue-specific TFs (50 in total). A total of 147 BTB genes were expressed in nine tissues, of which 82 were core TFs and 65 were classified into softcore and tissue specific. The majority of C2H2 TFs (111 out of 146) were commonly expressed in nine adult tissues, while a large number of homeobox TFs (78 out of 98) were preferentially expressed in several tissues. The distinct expression patterns of different TF families suggested their diverse regulatory functions in tissue development.

### 3.4. Sex-Related Transcription Factors in Crassostrea Oysters

**Figure 5:**
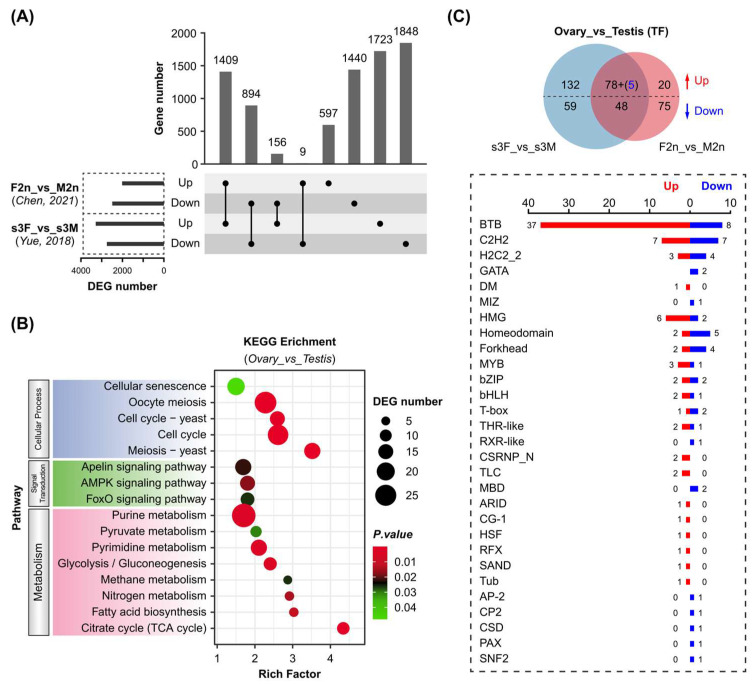
Sex-biased expression of transcription factors in Pacific oyster gonads. (**A**) Upset plot of differentially expressed genes (DEGs) between female and male gonads. DEGs were generated based on two gonad transcriptome datasets from published research as described in Materials and Methods. (**B**) KEGG pathway enrichment of gonadal DEGs. The shared 1409 up-regulated and 894 down-regulated DEGs in the two transcriptome datasets were used for enrichment analysis. (**C**) Differentially expressed TF genes in female and male gonads. Five TF genes marked in blue in the bracket were up-regulated in s3F_vs_s3M group but down-regulated in F2n_vs_M2n group. Female gonads (ovary) and male gonads (testis) were used as control and experiment groups, respectively [10,23].

The Pacific oyster has emerged as a good model for studying sex determination mechanisms. Previous research has been focused on the conserved sex-determining genes, such as *Dmrt1* [6], *Foxl2* [16], and the Sox gene family [20]. However, the information about other key TFs involved in *C. gigas* gonadal differentiation and development is still limited. In this study, sex-related TFs were identified by comparative transcriptome analysis of *C. gigas* female and male gonads. Two transcriptome datasets from previous research [10,23] were used to obtain differentially expressed genes (DEGs) between *C. gigas* female and male gonads (Supplementary Figure S3). Both studies used gonad samples in the mature stage, and a high correlation was observed within each group (Supplementary Figure S3A,D). However, their number of total and TF genes expressed in each group varied significantly. A total of 703 and 794 TFs were identified in s3F/s3M and F2n/M2n, respectively (Supplementary Figure S3B,E). These variations might result from dynamic regulation and relatively low expression levels of TFs (Supplementary Figure S3C,F). Therefore, the shared DEGs with the same regulation were screened between the two datasets, resulting in 1409 co-up-regulated and 894 co-down-regulated genes (Figure 5A). KEGG enrichment analyses of these 2303 DEGs indicated that female and male gonads differed significantly in cellular processes such as meiosis and cell cycle (Figure 5B). Interestingly, DEGs were obviously enriched in cellular metabolism pathways, including carbohydrate metabolism (glycolysis/gluconeogenesis, citrate cycle, pyruvate metabolism), lipid metabolism (fatty acid biosynthesis), and nucleotide metabolism (purine metabolism, pyrimidine metabolism). Furthermore, 78 up-regulated and 48 down-regulated TF genes were identified in both datasets (Figure 5C). Five TF genes were removed due to their opposite regulation in the two datasets. These 126 genes covered 29 TF families, in which BTB was the most abundant family (37 up-regulated and 9 down-regulated). It was not surprising to discover that *Dmrt1* and *Foxl2* were highly expressed in male and female gonads, respectively (Supplementary Table S5).

**Figure 6:**
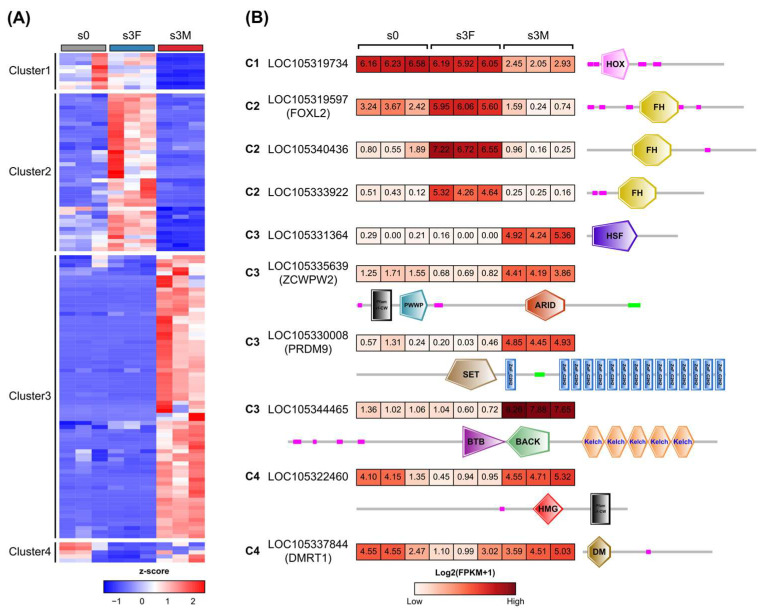
Dynamic regulation of sex-biased transcription factors during gonadal development. (**A**) Heatmap showing differential expression levels of sex-biased TFs during gonadal maturation. Z-score was generated using FPKM values. *C. gigas* gonad at the inactive stage was indicated as s0. (**B**) Representative TFs related to gonadal development and their DNA-binding domains. C1 to C4 corresponded to cluster1 to cluster4 in the heatmap.

Next, dynamic expression patterns of 126 sex-related TFs were investigated between the inactive stage and the mature stage (Supplementary Table S5). Three TFs were removed due to their low expression levels in gonads, including LOC105318699 (BTB), LOC117690080 (BTB), and LOC105331182 (THR-like). The sex-related TFs were divided into four clusters based on their expression levels in the inactive stage (s0) and mature stage (s3F, s3M), as shown in Figure 6A. Most of these TFs were up-regulated in female or male gonads in the mature stage compared to the inactive stage (clusters 2 and 3), demonstrating their essential roles in gonadal differentiation and development. Interestingly, *Dmrt1* and *Foxl2* were co-expressed in the inactivate stage (Figure 6B). It is consistent with the finding that the interplay between *Dmrt1* and *Foxl2* could determine the differentiation direction of bipotential gonads [16]. *Prdm9* (PR domain-containing 9) and *Zcwpw2* (zinc finger CW-type and PWWP domain-containing 2), two important genes involved in meiosis [28,29], were expressed at low levels in the female gonad, but were highly expressed in the male gonad. The testis-determining *SoxH* (LOC105319856) was expressed at high levels in the male gonad (Supplementary Table S5). It is worth noting that the functions of the most sex-related TFs remained unknown. Therefore, these results would provide a valuable resource for *C. gigas* sex-determining studies in the future.

**Figure 7:**
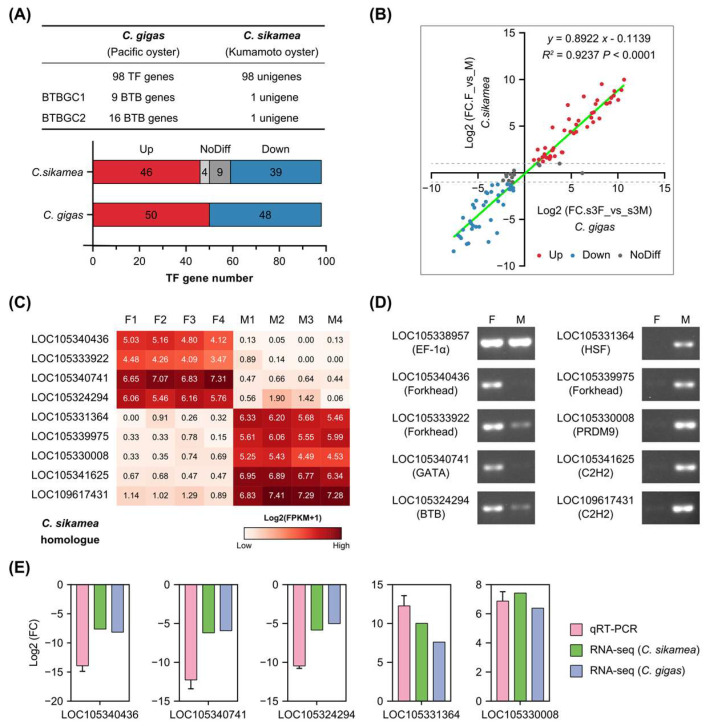
Highly conserved regulation of sex-related transcription factors in two Crassostrea species. (**A**) Comparison of sex-biased TFs between Pacific oyster (*C. gigas*) and Kumamoto oyster (*C. sikamea*). BTBGC, BTB gene cluster. (**B**) Linear regression analysis of fold change (FC) of sex-biased TFs between *C. gigas* and *C. sikamea*. (**C**) Expression levels of nine *C. sikamea* TFs in gonads based on FPKM values. (**D**) RT-PCR analyses of sex-biased TFs in *C. sikamea* gonads. F: female gonad, M: male gonad. (**E**) Quantitative real-time PCR (qRT-PCR) verification of sex-biased TFs in *C. sikamea* gonads. Fold changes (FCs) of five TFs were determined by qRT-PCR and compared to *C. sikamea* and *C. gigas* transcriptomic results. The Log2(FC) values are shown as mean with SD (standard deviation).

To determine whether these sex-related TFs were conserved in the Crassotrea genus, we compared differentially expressed TFs of *C. sikamea* gonad transcriptomes with *C. gigas*. *C. sikamea* transcriptomes were generated in our laboratory using mature female and male gonads (Supplementary Table S2). *C. sikamea* shares a close relationship with *C. gigas* [30]. Due to the lack of the *C. sikamea* genome during transcriptome sequencing, gonad transcriptomes were assembled de novo and used for DEG analysis. Homologues of 98 *C. gigas* TF genes were found in the *C. sikamea* transcriptome with one-to-one correspondence (Figure 7A and Supplementary Table S6). However, 9 BTB genes and 16 BTB genes corresponded to one *C. sikamea* unigene for each gene cluster. Further investigation demonstrated that these genes shared high sequence similarities in each cluster ( Supplementary Table S6). Thus, the two gene clusters were referred to as BTBGC1 and BTBGC2 in this study, respectively.

Furthermore, we compared the regulation of 98 conserved TF genes in the two Crassostrea species. Fold changes of the 98 genes showed linear correlation in *C. gigas* and *C. sikamea* (Figure 7B), indicating these sex-related TFs were co-regulated in the gonads of Crassostrea species. Four female-specific and five male-specific TFs were selected for reverse transcription PCR (RT-PCR) verification in *C. sikamea* gonads. Based on their FPKM values, these genes were predominantly expressed in the female or male gonad (Figure 7C). RT-PCR results were consistent with transcriptome analysis and confirmed their biased expression in the gonads (Figure 7D). Relative expression of five genes from five TF families was also examined by use of quantitative real-time PCR (qRT-PCR). The results showed that their fold changes were highly consistent with the transcriptomic analyses (Figure 7E). In summary, these sex-related TFs represent a conserved gene set that orchestrates gonadal differentiation and development in Crassostrea oysters.

### 3.5. Duplication of BTB Genes in Crassostrea Oyster Genomes

**Figure 8:**
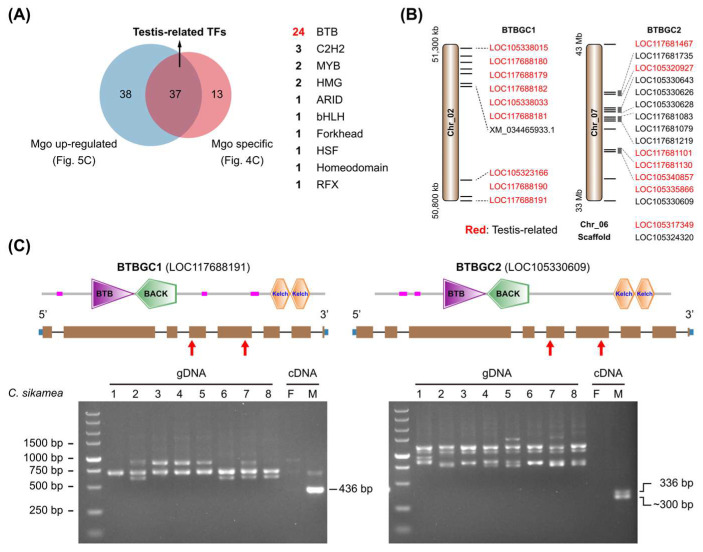
BTB gene duplication in Crassostrea genomes. (**A**) Screening of testis-related TFs. A total of 37 TFs were identified to be specifically expressed in Pacific oyster male gonads. (**B**) Chromosomal distribution of two BTB gene clusters (BTBGCs) in *C. gigas* genome. XM_034465933.1 was annotated as one transcript of LOC105323166 in GCF_902806645.1, but it should be an independent BTB gene. (**C**) PCR examination of BTB gene duplication in Kumamoto oyster. Two primer pairs were designed based on gene models in *C. gigas* and were used to investigate BTB gene duplication in *C. sikamea* genomes (gDNA, genomic DNA). Female and male gonad cDNA samples were used as controls. Red arrowheads indicated the position of primers.

A high proportion of male-gonad-specific (Mgo-specific) and male-gonad-up-regulated (Mgo-up-regulated) TFs belonged to the BTB family (Supplementary Table S4 and Figure 5C). Integration of these two datasets generated 37 TF genes that were believed to be specifically expressed in the male gonad (testis-related TFs), including 24 BTB genes (Figure 8A).

Further, it was found that most of the BTBGC1 and BTBGC2 members were included in the testis-related TFs (Figure 8B). BTBGC1 contained ten genes and was distributed in 0.5 Mb fragment on Chr_02. The transcript XM_034465933.1 was annotated as one isoform of LOC105323166, but it should be an independent gene. BTBGC2 contained 16 genes, of which 14 were distributed in 10 Mb fragment on Chr_07. BTB genes in each cluster had similar gene structures, high protein identities, and even similar promoter sequences (Figure 8C). It is no wonder that they were simultaneously up-regulated in the male gonad. Since there are four chromosome-level genomes of *C. gigas* in the NCBI database (https://www.ncbi.nlm.nih.gov/datasets/genome/?taxon=29159, accessed on 20 March 2025), we compared gene numbers of BTBGC1 and BTBGC2 in different assemblies (Table 2). BTBGC1 showed significant gene number variations in different *C. gigas* genomes, from 5 to 10 copies. BTBGC2 showed similar distribution patterns in the genome, with 13 (or 14) genes on a single chromosome, and 1 gene each in two other chromosomes. We also examined gene numbers in other Crassostrea species, including *C. angulata*, *C. ariakensis*, *C. hongkongensis*, and *C. nippona*. Notably, the gene numbers of two BTBGCs were similar in *C. gigas* and *C. angulata*, two closely related species. However, they varied significantly in *C. ariakensis*, *C. hongkongensis*, and *C. nippona*. The latter three species had many fewer gene copies than the former two, suggesting BTB gene duplication might be involved in the evolution of the Crassotrea species.

We further investigated the expression of the two BTBGCs in *C. sikamea* gonads. RT-PCR results showed that the two BTBGCs were specifically expressed in the male gonad (Figure 8C), which was consistent with transcriptome analysis (Supplementary Table S6). A chromosome-level genome of *C. sikamea* was published recently [31]. A total of five genes in BTBGC1 and eight genes in BTBGC2 were found in the *C. sikamea* genome (Supplementary Table S7). PCR amplification of genomic DNA samples showed diverse bands and sizes among different *C. sikamea* individuals (Figure 8C), suggesting high variations of the two BTBGCs in the genomes. Future studies are required to clarify their gene number variations in Crassostrea oysters and their roles in male gonad development.

## 4. Discussion

Bivalves constitute the second-largest class in the phylum Mollusca [32]. The Pacific oyster *C. gigas* (Bivalvia: Ostreidae) has been widely used for sex determination studies in the past decades. Deciphering the transcriptional regulation of TFs in *C. gigas* sex determination and gonadal differentiation requires a comprehensive knowledge of TF families and numbers in the genome. In this study, a total of 1167 TF genes were identified in the *C. gigas* genome (Figure 1A). About 3.72% (1167/31,371) of *C. gigas* protein-coding genes are predicted to encode TFs, far below the 6~9% of TFs in the human genome [12]. Although some TFs might not be included in this research due to high sequence variation and/or incorrect genome annotation, this is the first report of genome-wide identification of TF families in the Pacific oyster. This research will not only facilitate our understanding of TF genes in the Crassostrea species but also provide fundamental information for TF identification in other bivalves.

### 4.1. Distinct Expression Patterns of TF Families in Pacific Oyster

It should be noted that *C. gigas* genome annotations are far from perfect. The current reference genome of *C. gigas* in NCBI (accession number GCF_963853765.1) contains many fewer scaffolds than GCF_902806645.1 (19 versus 226). However, some important genes are missing in the new genome assembly, such as glucose-6-phosphate dehydrogenase (G6PD). We also examined TF families based on the new genome and obtained similar but not identical gene numbers in each family. Therefore, the older version GCF_902806645.1 was used in this study to systematically investigate TF genes and their expression patterns.

There are 73 TF families in *C. gigas*, in which the C2H2 zinc finger is the largest family (Figure 1B). It is consistent with the largest number of C2H2 TFs in human [33]. Homeobox genes are well known for their sequential and cell lineage-specific expression during larval development [14,34]. Based on FPKM values ≥ 1, about three-quarters of TF genes (918/1167) were expressed in nine adult tissues (Figure 4B and Supplementary Table S4). About 59.04% of these TF genes were expressed in all the tissues (core TF genes), which might play fundamental roles in tissue development. C2H2 and homeobox are two major TF classes in *C. gigas* (Figure 1B); however, they have different expression preferences in adult tissues. About 76.03% of C2H2 TFs (111/146) were ubiquitously expressed in adult tissues, while 79.59% of homeobox TFs (78/98) were selectively expressed in particular tissues (Figure 4C). These expression divergences are also observed in human C2H2 and homeobox TFs [12], suggesting conserved roles of the two TF classes in tissue differentiation and maintenance.

### 4.2. Sex-Related TFs in Crassostrea Oysters

It has long been recognized that sex-related TFs serve as essential regulators in sex determination and gonadal differentiation [35]. Despite several sex-determining TFs having been reported in *C. gigas* [6,15,20], they are mainly identified based on homology to well-characterized genes in model organisms (human, mouse, fruitfly, et al.). In this study, a gene set of 123 TFs was screened based on DEGs between *C. gigas* female and male gonads (Figure 5C). This TF gene set includes *Dmrt1*, *Foxl2*, and Sox genes specifically expressed in the female or male gonad (Supplementary Table S5), as described in previous studies [16,19,20]. These results suggest high reliability of this TF gene set in coordinating oyster gonadal development. All seven Sox genes reported in the previous research [20] were included in the HMG family (Supplementary Table S3). Moreover, our research identified 33 genes with the conserved HMG domain, which would enable the thorough investigation of Sox genes in *C. gigas*. Human TFs usually function in complexes through protein–protein interactions [36]. DMRT1 is regarded as a pioneer TF that could interact with SOX9 and alter the chromatin structure to let other TFs bind their target genes [37]. It will be of great interest to investigate *C. gigas* TF interaction networks and their binding sites in target genes during gonadal development in future studies.

### 4.3. Cellular Processes Potentially Regulated by Sex-Related TFs in Oyster Gonads

PRDM9 and ZCWPW2 play crucial roles in vertebrate meiosis by binding at the recombination hotspot during the zygotene stage (prophase of meiosis I) [28,29]. KEGG enrichment of DEGs suggested that *C. gigas* female and male gonads differed significantly in meiotic gene expression (Figure 5B). *Prdm9* and *Zcwpw2* were highly expressed in the mature male gonad, but showed extremely low levels in the mature female gonad (Figure 6B). A similar expression pattern was also observed in three synaptonemal complex protein (SYCP) genes, indicating different meiosis processes between female and male germ cells, especially in the synapsis stage. It is consistent with the observation that oyster oocytes are generally arrested in the prophase or metaphase stage of meiosis I until fertilization [38]. How the other TF (or non-TF) regulators collaboratively interact with PRDM9 and ZCWPW2 and contribute to the meiosis of spermatocytes and oocytes needs further investigation.

BTB zinc finger is the second-largest TF family in *C. gigas* (Figure 1B). BTB is a conserved domain that mediates protein–protein interactions and participates in diverse cellular functions [39,40]. BTB proteins are well characterized as substrate adaptors in the cullin-RING ligase (CRL) E3s that determine substrate specificities in the ubiquitin–proteasome system [41]. It is interesting that two BTB gene clusters were specifically expressed in the male gonad (Figure 8C and Supplementary Table S5). In addition, gene numbers in each cluster varied significantly among six Crassostrea species (Table 2). The large number of BTB genes in *C. gigas* might be the consequence of gene duplication. To date, there is no publication about BTB genes in oysters. The functions of BTB proteins in oyster gonadal development remain to be investigated.

## 5. Conclusions

Collectively, this research unveils the first genome-wide identification of TF families in the Pacific oyster *C. gigas*. Our results will provide a fundamental resource for TF investigation in oysters and other bivalve species. Importantly, the sex-related gene set of 123 TFs will provide valuable information for future studies aimed at examining sex determining and gonadal differentiation mechanisms of oysters.

## Figures and Tables

**Table 1:** List of primers used in this study.

Note	Gene ^1^	Sequence (5′-3′)	Purpose	Expected Size (bp)
EF-1α	LOC105338957	CAACATCACCACTGAGG	RT-PCR	475
		AACCTCCTTGATGACAC		
		AGGCTGACTGTGCTGTGTTG	qRT-PCR	278
		GTTATCTCCGTGCCATCCAG		
Forkhead	LOC105340436	TCTGAACAACCAGGACC	RT-PCR	448
		GAACTTGGCATACATCG		
		GAGTCTGGAATCGACATCAC	qRT-PCR	229
		GTTGGCGTTCATTTGGTAGG		
Forkhead	LOC105333922	GCTAAGTGACATCTACC	RT-PCR	403
		GTGATGCGATGTAATGG		
GATA	LOC105340741	TTTGACTCCTCGAACAC	RT-PCR	431
		GTACCGTCATCTGCATC		
		GAAGTCAAGAACAGGAACTG	qRT-PCR	268
		TGATTCCACACGCATTACAC		
BTB	LOC105324294	AGAAGCCAGCAATCTAC	RT-PCR	463
		AACGAAGTGGAATCAGG		
		ACGTCCTGTTTCTTGTTGGA	qRT-PCR	238
		ACCTCAATCGTGACCTTGTC		
HSF	LOC105331364	ACAGATGCTATTGGATG	RT-PCR	564
		CAGAAGGAATCACGTTG		
		ACAGACCTTTCCAAACAACG	qRT-PCR	229
		ATCCTGGCAAAAGCATCCTC		
Forkhead	LOC105339975	GTGCAAACCATTGTAGC	RT-PCR	403
		CCTGATCTCTATTCTCC		
PRDM9	LOC105330008	GAAGCACACGAAACAGG	RT-PCR	348
		CAGCCACTCATAACTAC		
		GAAGAAGCACACGAAACAGG	qRT-PCR	222
		ATACCACACCAACAGCTCAG		
C2H2	LOC105341625	TGAGCTGAGTCTGTCTG	RT-PCR	392
		TGACAGAGTGAATAAGC		
C2H2	LOC109617431	GTTGAAATGCCTTCTCC	RT-PCR	463
		GGAGGTTTACGATGTGG		
BTBGC1	LOC117688191	CACTGAGCAGGTTGATG	RT-PCR	436
		GGTCTGCTCCCAATCTC		
BTBGC2	LOC105330609	CTAATGACTGAAGATGC	RT-PCR	336
		GTCCTGCATTCGATCTC		

^1^ Shown as *C. gigas* homologues.

**Table 2:** Gene number variations of two BTBGCs in Crassostrea species.

Species	Genome Assembly	BTBGC1	BTBGC2
*C. gigas*	GCF_902806645.1	NC_047560.1 (10)	NC_047565.1 (14), NC_047564.1 (1), NW_022994836.1 (1)
*C. gigas*	GCF_963853765.1	NC_088853.1 (5)	NC_088854.1 (13), NC_088855.1 (1), NC_088857.1 (1)
*C. gigas*	GCA_025765685.3	CM056677.2 (10)	CM056679.2 (13), CM056680.2 (1), CM056684.2 (1)
*C. angulata*	GCF_025612915.1	NC_069112.1 (6)	NC_069117.1 (14), NC_069116.1 (1), NC_069111.1 (1)
*C. angulata*	GCA_025765675.3	CM056667.2 (6)	CM056669.2 (14), CM056670.2 (1), CM056674.2 (1)
*C. ariakensis*	GCA_020458035.1	CM035811.1 (10)	CM035815.1 (6), CM035814.1 (1)
*C. ariakensis*	GCA_040114485.1	CM079021.1 (9)	CM079018.1 (3), CM079019.1 (1)
*C. hongkongensis*	GCA_016163765.1	WFKH01011926.1 (5)	WFKH01011581.1 (2)
*C. nippona*	GCA_033439105.1	CM065917.1 (2)	CM065918.1 (6)

## Data Availability

Accession numbers of transcriptome data used in this study have been listed in Supplementary Table S2.

## Supplementary Materials

The following supporting information can be downloaded at https://www.mdpi.com/article/10.3390/genes16050513/s1, Figure S1. Chromosomal distribution of four transcription factor families in Pacific oyster genome. (A) Circos plots showed C2H2, BTB, Homeobox, and THAP gene densities from outer to inner circles. The sliding window size was set as 1 Mb. Gene numbers of each TF family on ten chromosomes were listed below the circos plots. (B) Chromosomal localization of BTB genes. Figure S2. Alternative splicing events in Pacific oyster transcription factors. (A) Summary of *C. gigas* TF genes with alternative splicing. The number indicated TF genes with alternative splicing based on genome annotation. ZF, zinc finger; *HOX*, homeobox, NR, nuclear receptor. (B) Sashimi plots of two *C. gigas* TF genes with different spliced transcripts. ES, exon skipping; AT, alternative terminator. Figure S3. Comparative transcriptome analysis of Pacific oyster female and male gonads. (A,D) Pearson correlation coefficient of six gonad transcriptomes. (B,E) Comparison of total and TF genes expressed in female and male gonads. (C,E) Expression levels of non-TF and TF genes in female and male gonads. Table S1. Overview of Pacific oyster genome annotation. Table S2. Summary of Pacific oyster transcription factors. Table S3. Transcriptomic data used for gene expression analyses. Table S4. Expression patterns of Pacific oyster transcription factors in nine adult tissues. Table S5. FPKM values of sex-biased transcription factors in Pacific oyster gonads. Table S6. Comparison of sex-biased transcription factors in Pacific oyster (*C. gigas*) and Kumamoto oyster (*C. sikamea*) gonads. Table S7. Chromosomal localization of two BTBGCs in the Kumamoto oyster (*C. sikamea*) genome.

## Abbreviations

The following abbreviations are used in this manuscript:

TF	transcription factor
BTBGC	BTB gene cluster
DEG	differentially expressed gene
ES	exon skipping
A3SS	alternative 3′ splicing site
A5SS	alternative 5′ splicing site
IR	intron retention
MXE	mutually exclusive exons
AT	alternative terminator
Amu	adductor muscle
Dgl	digestive gland
Mgo	male gonad
Hem	hemolymph
Fgo	female gonad
Gil	Gill
Lpa	labial palp
Mou	mantle outer region
Min	mantle inner region
