# Supplementary figures and images for: Integrated Genomic and Transcriptomic Analysis Reveals a Transcription Factor Gene Set Facilitating Gonadal Differentiation in the Pacific Oyster Crassostrea gigas
Source: Genes (Basel). 2025 Apr 28;16(5):513. doi: 10.3390/genes16050513 (PMC12111275; doi:10.3390/genes16050513)

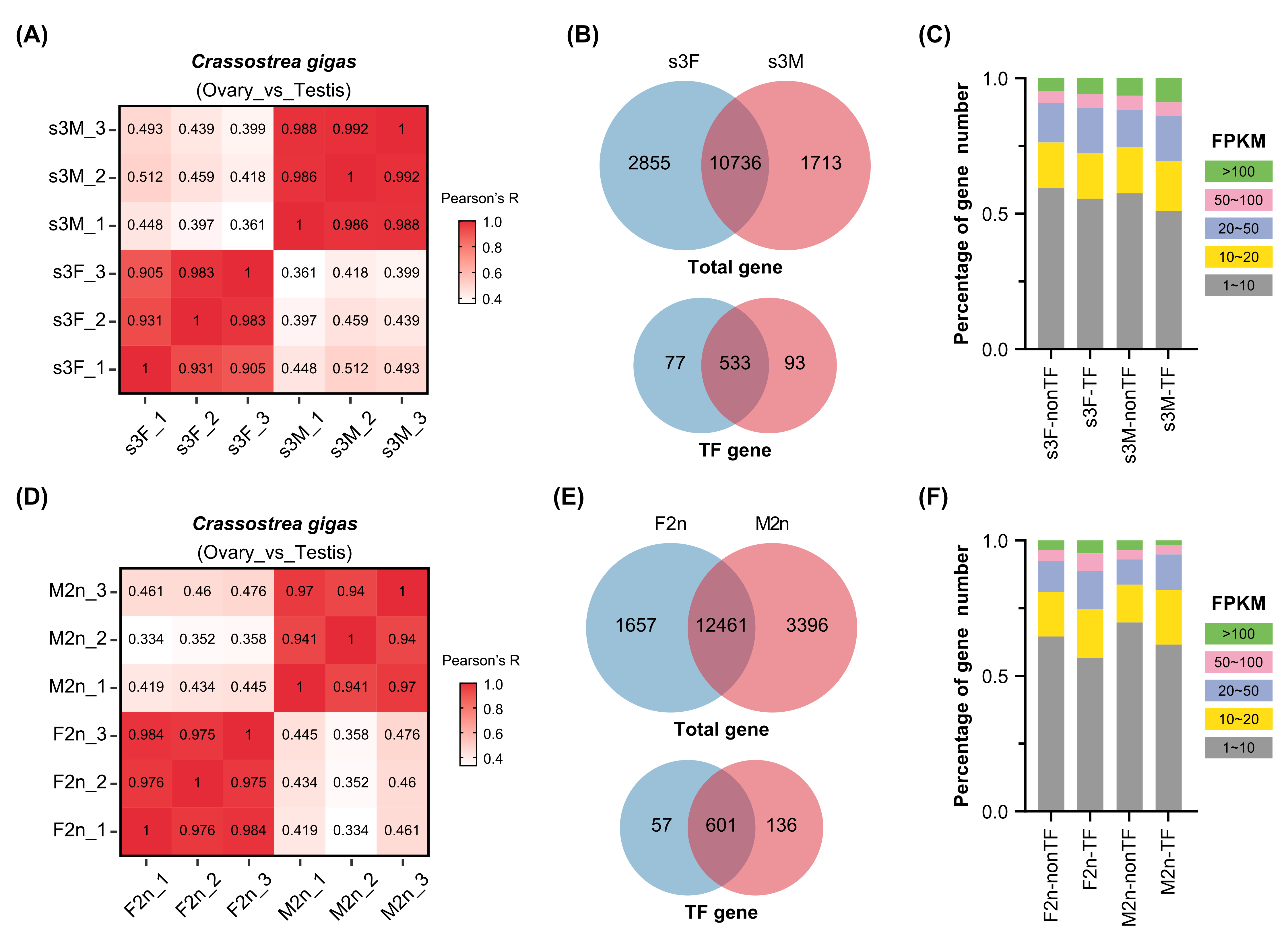

Supplement: Supplementary file 1 [file genes-16-00513-s001.zip › Fig.S3.tif]
